# Implementation of a patient-facing genomic test report in the electronic health record using a web-application interface

**DOI:** 10.1186/s12911-018-0614-x

**Published:** 2018-05-30

**Authors:** Marc S. Williams, Melissa S. Kern, Virginia R. Lerch, Jonathan Billet, Janet L. Williams, Gregory J. Moore

**Affiliations:** 1 Genomic Medicine Institute, Geisinger, 100 North Academy Avenue, Danville, PA USA; 2 Center for Pharmacy Innovation and Outcomes, Geisinger, Danville, PA USA; 3 Institute for Advanced Application, Geisinger, Danville, PA USA

**Keywords:** Genomics, Medical informatics applications, Electronic health records, Patient-centered care, Patient access to records, Communication

## Abstract

**Background:**

Genomic medicine is emerging into clinical care. Communication of genetic laboratory results to patients and providers is hampered by the complex technical nature of the laboratory reports. This can lead to confusion and misinterpretation of the results resulting in inappropriate care. Patients usually do not receive a copy of the report leading to further opportunities for miscommunication. To address these problems, interpretive reports were created using input from the intended end users, patients and providers. This paper describes the technical development and deployment of the first patient-facing genomic test report (PGR) within an electronic health record (EHR) ecosystem using a locally developed standards-based web-application interface.

**Methods:**

A patient-facing genomic test report with a companion provider report was configured for implementation within the EHR using a locally developed software platform, COMPASS™. COMPASS™ is designed to manage secure data exchange, as well as patient and provider access to patient reported data capture and clinical display tools. COMPASS™ is built using a Software as a Service (SaaS) approach which exposes an API that apps can interact with.

**Results:**

An authoring tool was developed that allowed creation of patient-specific PGRs and the accompanying provider reports. These were converted to a format that allowed them to be presented in the patient portal and EHR respectively using the existing COMPASS™ interface thus allowing patients, caregivers and providers access to individual reports designed for the intended end user.

**Conclusions:**

The PGR as developed was shown to enhance patient and provider communication around genomic results. It is built on current standards but is designed to support integration with other tools and be compatible with emerging opportunities such as SMART on FHIR. This approach could be used to support genomic return of results as the tool is scalable and generalizable.

## Background

Precision Medicine with an emphasis on the incorporation of genomic information into the clinic has been identified as a priority for research in the United States [1].

Genetic disorders, while individually rare, are collectively common. It is estimated that there are over 6800 rare and ultra-rare disorders, many of which are genetic affecting approximately 30 million Americans [2]. The challenge for patients and their providers is having ready access to the information that is necessary for appropriate management and coordination of care. When faced with a patient with a genetic condition they have not previously encountered, knowledge and resources are extremely limited. This often puts patients and families in the position of attempting to become the ‘expert’ in the specific disease via an unguided internet search process which can lead to uncomfortable and sometimes adversarial encounters with the medical system.

One potential solution to lower the barriers experienced by providers caring for patients with genetic conditions is using fully functional electronic health record systems (EHR) [3–5]. The capabilities provided by such EHRs, particularly through knowledge management systems and clinical decision support systems, have been demonstrated to significantly improve process outcomes, although the evidence of impact on health outcomes is less robust [6].

While these techniques have mostly faced toward providers, similar approaches could be used to provide information directly to patients and families. A recent study conducted at 3 healthcare organizations, including Geisinger, studied the impact of opening patient access to their provider notes through a secure patient portal (OpenNotes) [7]. Using a quasi-experimental trial design, several significant differences were noted when comparing the pre- and post-intervention surveys including patients reporting that OpenNotes, “…helped them feel more in control of their care” and “…increased medication adherence:”. A recent article noted that patient-facing applications could enable meaningful use objectives promoted by the Office of the National Coordinator of Health Information Technology (ONCHIT) [8]. This approach is also endorsed by the recent report from the National Academy of Medicine (formerly the Institute of Medicine), Improving Diagnosis in Health Care [9] which highlights the importance of patient empowerment.

One area that has received less emphasis is the role of the laboratory report in communicating information to patients. Haga et al. [10] propose four revisions to current genomic test reports that could improve accessibility for patients. These include, “…1) inclusion of an interpretive summary section, 2) a summary letter to accompany the lab report, 3) development of a patient user guide to be provided with the report, and 4) a completely revised patient-friendly report.” In genomic medicine, direct-to-consumer companies have emerged that provide results of tests to consumers without the intermediary of a health care professional. While many of the results are non-health related (e.g. ancestry) some companies are returning predictive information that could have an impact on an individual’s health. The positive and negative outcomes of providing this information directly to consumers have not been rigorously studied.

Our group has previously reported on the design and testing of linked patient- and provider-facing genomic test reports [11, 12]. These reports are not meant to replace the laboratory report, but to provide additional information to guide interpretation and clarify clinical recommendations that result from the testing. As such they are compatible with the recommendation from Haga to create a “a patient-friendly report” [10]. A comparative effectiveness study [13] that compares the use of these reports to standard of care communication has recently concluded and demonstrates qualitative improvement in communication and satisfaction.

The purpose of this paper is to present the technical aspects of development and deployment of the first patient-facing genomic test report (PGR) within an EHR ecosystem using a locally developed web-application interface.

## Methods

A PGR was developed using a mixed-methods formative approach which has been previously reported [11]. The PGR includes content that was considered important by patients and their families. The content is vetted for readability through a review process that includes content experts, non-genetic providers and patients and is formally tested to meet accepted literacy standards. The PGR was developed using paper mock-ups. Once the final format was chosen, this was converted to an electronic format that could be displayed through an interface such as a patient portal, but could also be printed to provide a hard copy. A similar process was used to create a provider version of the PGR that was designed to complement the PGR [12]. These two reports were then submitted to the COMPASS™ development team for implementation.

COMPASS™ is a software platform developed at Geisinger intended to work as an add-on to EHRs. Its main purpose is to manage secure data exchange, as well as patient and provider access to patient reported data capture and clinical display tools utilized within Geisinger. It is intended to improve patient engagement in their care and enhance patient-provider communication.

COMPASS™ is built using the ASP.Net framework where the presentation layer (User Interface) implements web development languages and libraries including HTML, CSS, JavaScript, JQuery, AngularJS, and Angular2. The back-end (Code Behind, Logic, Data Interactions) is coded using C# and utilizes SQL Data Adapters, SOAP Web Services, WebAPIs, and Teradata Data Adapters to facilitate data transfers. The COMPASS™ data model utilizes MS SQL Server but it is not limited to only utilizing SQL Server for interacting with data as it can consume data from web services or any standard database platform through its Software as a Service (SaaS) approach. Through this, users can either pass data to the COMPASS™API for use or COMPASS™ can access common database platforms and consume/display appropriate data. It is designed so that any content can be coded to be accessible on any other resource. This allows content in COMPASS™ to be agnostic from its own server/resource perspective.

COMPASS™ is integrated with Geisinger’s primary EHR (Epic) on several levels. The EHR interacts with COMPASS™ by launching an external web browser via a specific encrypted URL. The current implementation within Epic is a visit navigator link which contains required encrypted parameters for authentication. COMPASS™ is fed by real-time data feeds from the EHR to correctly determine project inclusion/exclusion and display correct and current data to users. Currently, the real time feeds that COMPASS™ utilizes are custom and standard data extractions that interface with Epic. COMPASS™ is also integrated with patient portals (currently MyGeisinger which is a MyChart implementation) so patients can use the application when off-site from the health system network. As with the EHR, COMPASS™ interacts with the portal by launching an external web browser via an encrypted URL.

**Fig. 1:**
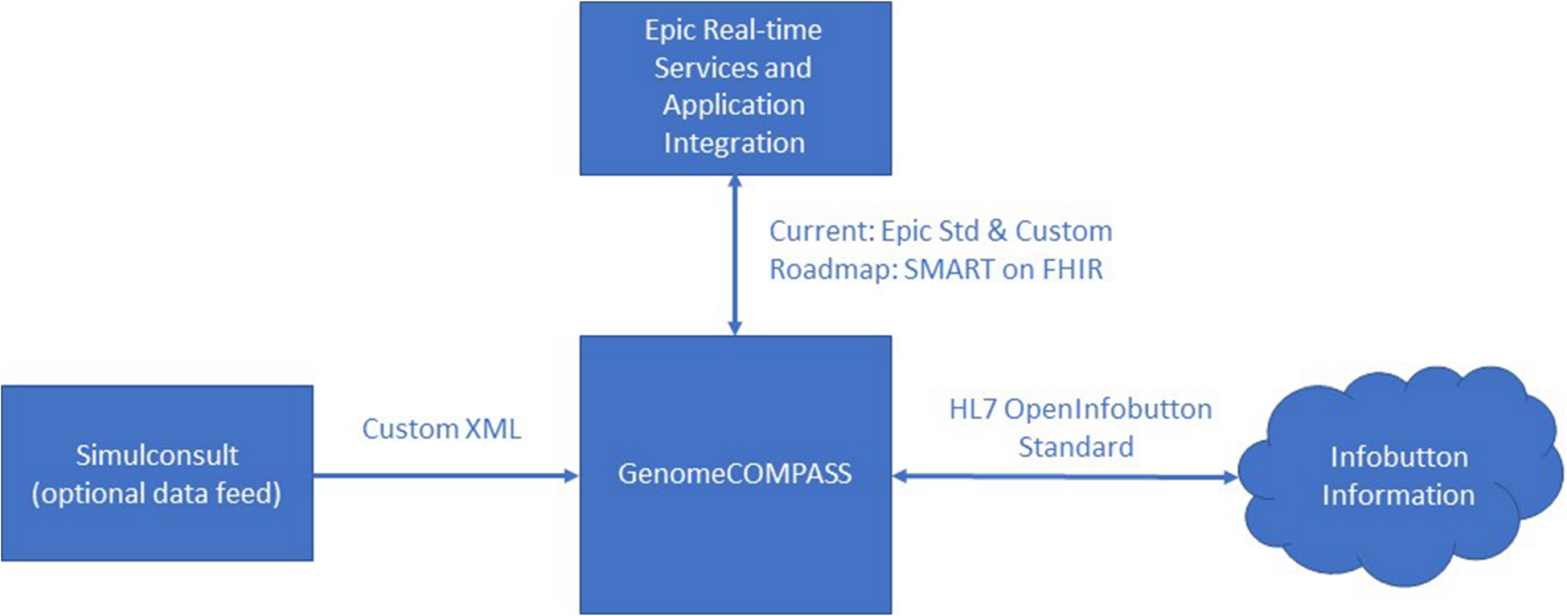
COMPASS™ Data Flow. A diagram of the data flows for COMPASS™. Note that the EHR data flow is in the process of moving to SMART on FHIR which should increase the generalizability of the tool in different EHR ecosystems

An authoring application was created within COMPASS™ to assist geneticists and genetic counselors with building coordinated and complementary patient and family-facing and provider-facing reports of the patient’s test results. The authoring application includes areas to create and store generic genetic content for the reports that is not readily available from other sources. The authoring environment contains reusable content fields (including inheritance patterns, glossary terms and gene templates that create gene-specific content that can be used for all reports that return results related to the gene) that can be selected to populate patient and provider reports. The environment tracks versioning of the information created within the authoring application—an essential element given the rapidly changing nature of knowledge in genomics that can have implications for liability. The ecosystem of the PGR report is presented in Fig. 1.

**Fig. 2:**
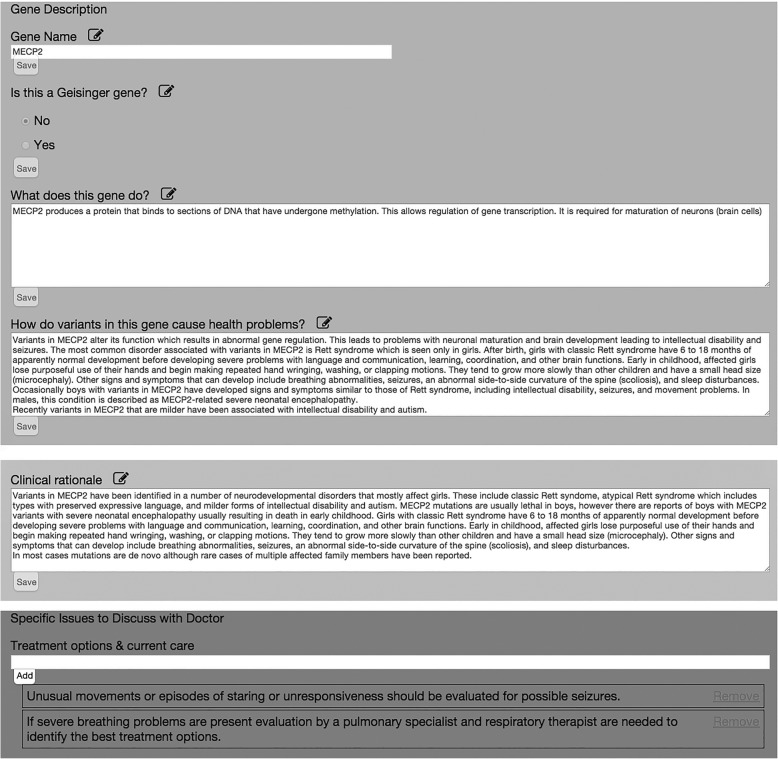
The generic gene template**.** An excerpt of the *MECP2* gene template from the COMPASS™ authoring tool

A patient report template is used by the geneticist/counselor to pull information together to create the patient specific reports. Through COMPASS™, the patient report template interfaces with various data sources in addition to the database storing the information generated from the generic gene template (Fig. 2), inheritance pattern template, and glossary term template. One specific tool, the SimulConsult® Genome-Phenome Analyzer (SGPA) [14] has been used to provide information to the report. A local API is called when a user finishes creating the content for the custom report in the authoring environment. The author then opens the Epic record for the patient and launches the SGPA from the encounter navigator. In the SGPA, patient information is combined with the genomic sequence information and a gene associated with the clinical features along with a variant in that gene are selected by the author. This ‘closes the loop’ by associating the gene and variant with the specific patient. The author then clicks the “Send XML Report” button in the SGPA which sends SimulConsult® content back to the authoring environment. A custom parser developed to support the project, in combination with the SimulConsult® Web API loads the data into the “PatientReports” table in the Genomics database. The final patient-specific report is available for review and publication at which time it appears in the MyGeisinger patient portal, and is accessible to clinicians in the patient’s encounter navigator through a local Web Apps URL. One specific example of SGPA content that was highly valued by both patients and providers was the prognosis table. This prognosis table, which summarizes medical issues associated with a condition the likelihood that the issue will develop and when in the life of the patient they are likely to appear, is automatically generated by the SGPA [14]. The manual creation of such a table would require a tremendous investment of time and expertise. While not required for the authoring process, use of the SGPA improves the workflow and reduces the time needed to author a report. Upon selection and verification of the content, the report content is published from the authoring application and made available to patients and their families and providers electronically in COMPASS™.

## Results

**Fig. 3:**
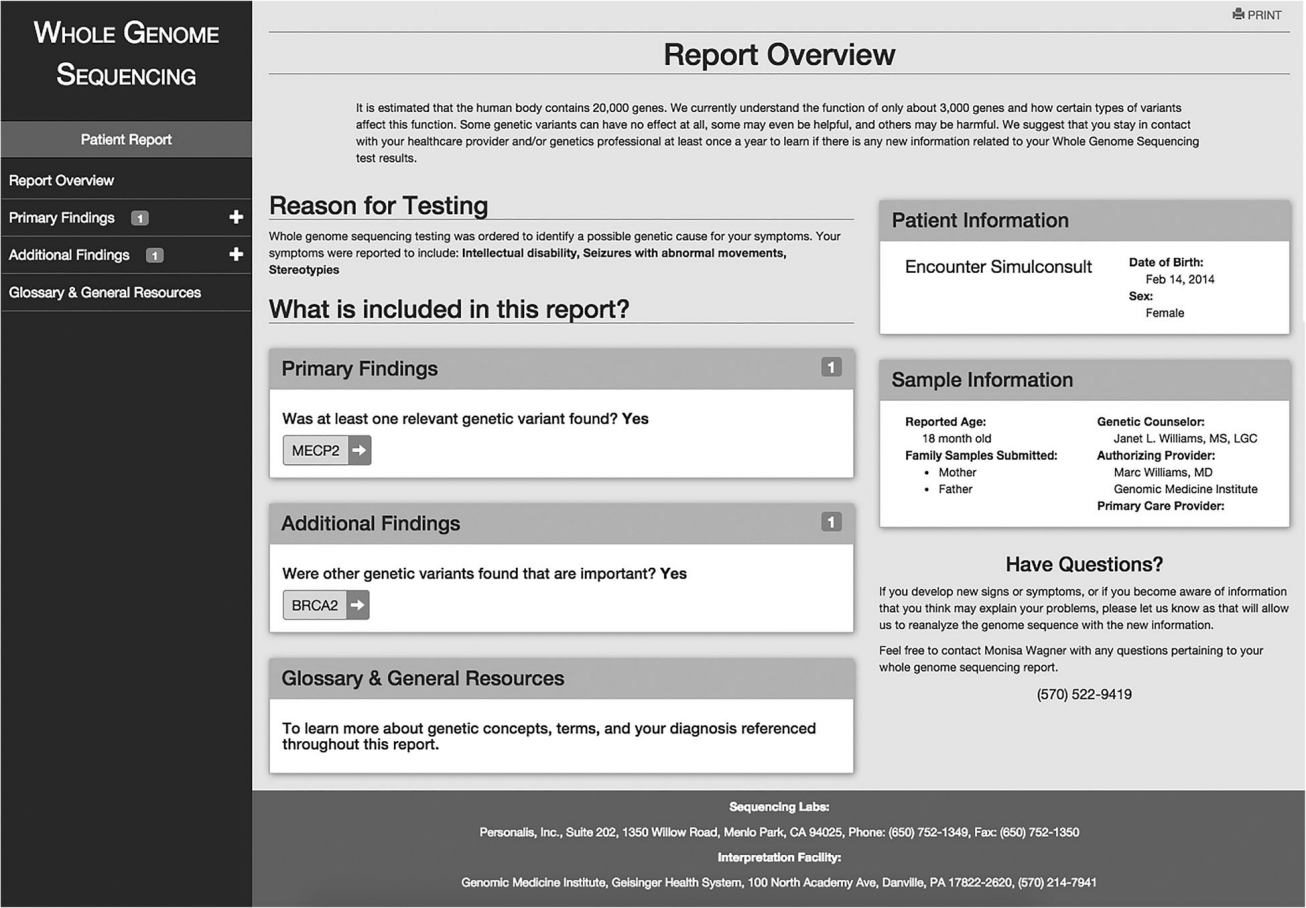
The published patient report. Overview. Shows the report overview which includes demographic information, a description of the reason for testing, primary and additional findings, and contact information

**Fig. 4:**
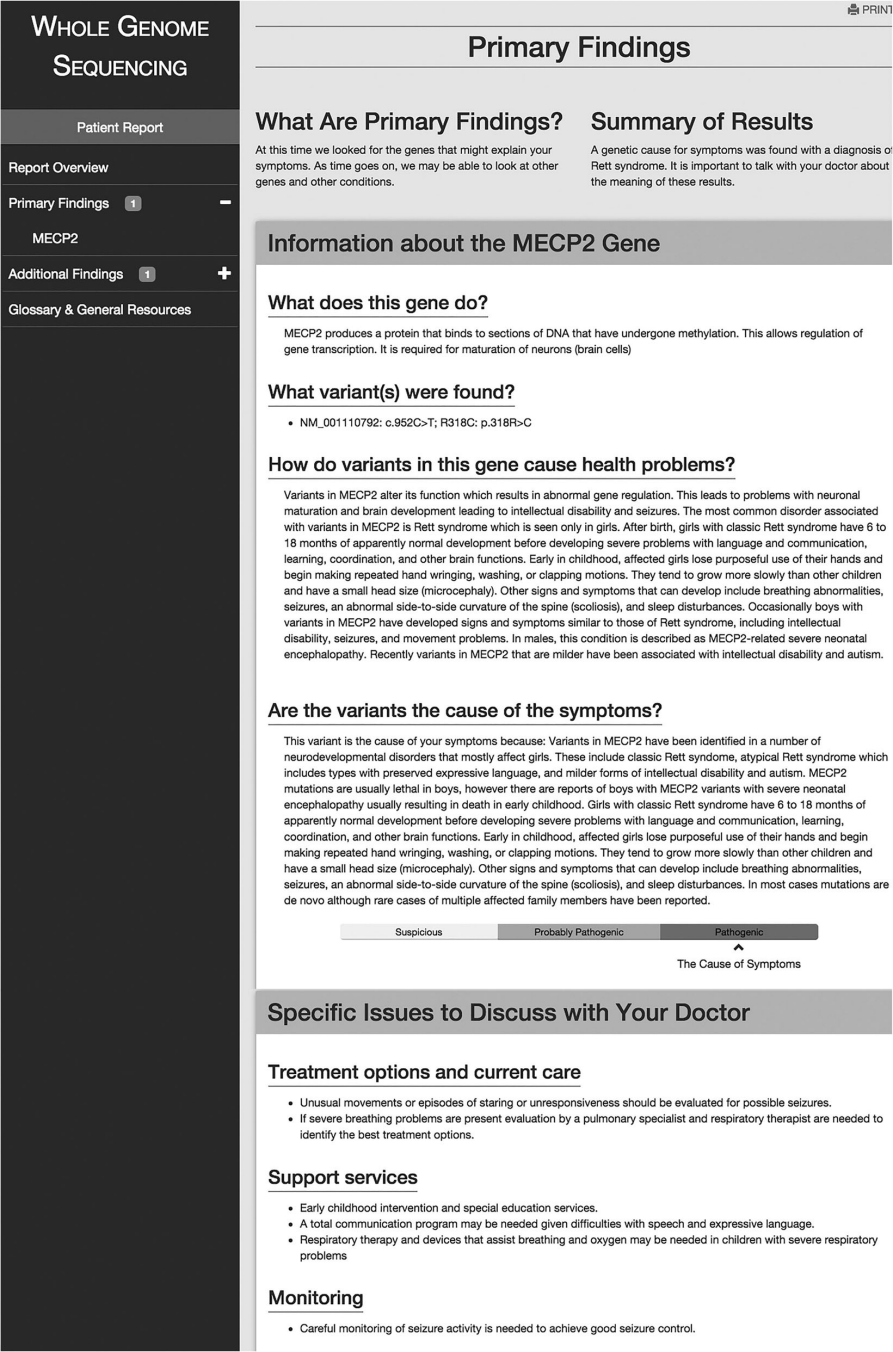
The published patient report: Primary finding detail. Shows the first portion of the report on the primary finding, in this case a pathogenic variant in the *MECP2* gene

After the PGR is generated in the authoring application of COMPASS™, the author will publish the report so that it is available to patients and their authorized representatives (usually family members). Patients and their families can access the report electronically through a link out to COMPASS™ from the patient’s MyGeisinger portal or prior to a visit in the clinic during the nurse rooming process in Epic (Fig. 3). The PGR is also designed so that a hard copy can be printed—a requirement that was requested by participants in the formative study. The report is written and laid out in a manner based on input from the interviews and focus groups that reflects the preferences of these end users. It presents the reason for genetic testing, information about the gene(s), variant(s) and associated diagnoses, prognosis, information for other family members, a glossary of genetic terminology, and resources to learn more such as disorder-specific support groups and sites designed for lay persons like Genetics Home Reference (Fig. 4). To promote engagement and empowerment, information is also provided about possible clinical trials relevant to the disorder (clinicaltrials.gov) and services such as GenomeConnect that support patient/family entered data to facilitate networking and research. Full details about the report are published elsewhere [11].

**Fig. 5:**
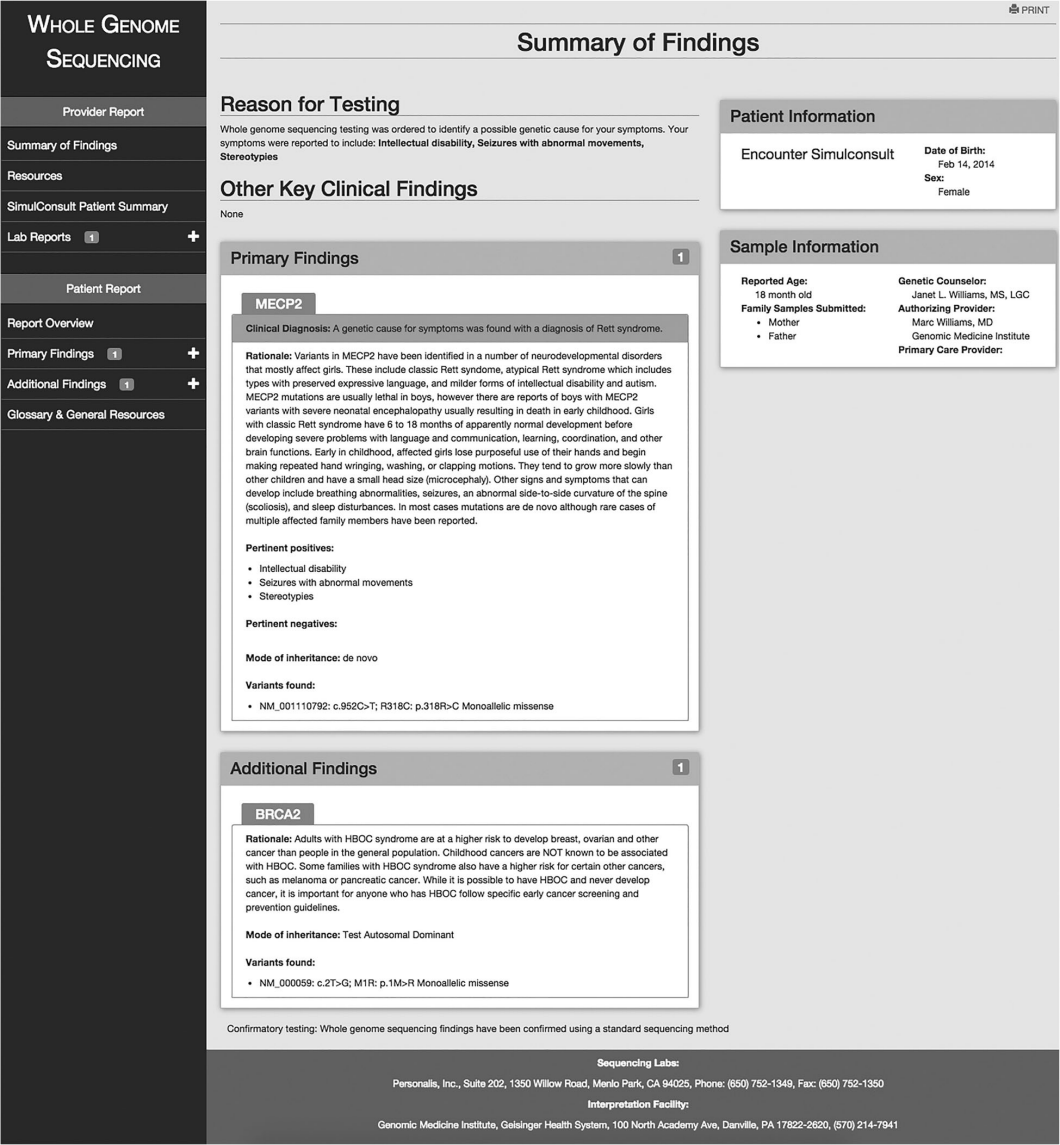
The published provider report: Summary. This shows the provider view of the summary of findings. As with the patient report, the provider can drill down for additional information on both the primary and additional findings for the patient. In addition, the provider can link to the SimulConsult® patient summary, access the report issued by the testing laboratory and can view (but not edit) the full content of the patient report

The provider-facing report (Fig. 5) is also made available at the point of care to primary care and genetic specialty providers who have access to COMPASS™ in Epic. COMPASS™ is embedded in the provider’s workflow in Epic in the visit navigator section and is designed to either be a manually or automatically launched depending on the clinic site’s needs. This report does not replace the actual laboratory report which is still scanned into the patient record (and is accessible through a link from the provider-facing report). It is meant to supplement the laboratory report to assist providers as they interpret the results and use them to guide care. This draft report was presented to providers and input from the providers was used to modify the draft [12]. The provider report includes standard information such as demographics, clinical data and testing information. The report highlights the indication for genetic testing, key findings, primary and secondary diagnoses and variant information, any genes that were found that require more research before conclusions can be drawn, a glossary of genetic terminology, and provider focused resources to learn more such as GeneReviews and Online Mendelian Inheritance in Man. The providers requested access to the PGR so that they are aware of information provided to the patients and their families. Access to the PGR is through a link within the provider report.

One additional enhancement that was added after the final reports were developed was the inclusion of infobuttons. Infobuttons are context-sensitive links embedded within information systems which allow easy retrieval of relevant information. They use contextual information about the patient, user, clinical setting, and EHR task to anticipate providers’ information needs and provide links to online clinical resources that may meet these information needs. While primarily directed to providers, there are emerging use cases that include infobuttons in patient-facing applications. Our enhancement took advantage of the emergence of OpenInfobutton, an open source suite of Web services that enable infobutton capabilities within EHR systems [15]. Use of this open source standard and its supporting resources allowed incorporation of the functionality into the PGR with minimal effort from the development team using the SaaS functionality in COMPASS. This was a high value addition to the report given that the Clinical Genome resource, which aggregates genetic and genomic information from a variety of sources, is accessible through OpenInfobutton [16].

## Discussion

The formative work that preceded development of the tool identified the need for such a tool as well as the elements that were needed for inclusion. The prototype underwent beta testing in the EHR and patient portal environments to identify and fix any technical problems that interfere with the tool’s access, performance and usability. The tool was deployed as part of a prospective randomized comparative effectiveness trial [13]. This trial used participants in a clinical research project that is using whole genome sequencing to determine the cause of undiagnosed intellectual disability. The trial was recently completed and confirmed that the report improved satisfaction, engagement and enhanced communication compared to standard of care for those participants where a causal variant was found. Qualitative evaluation identified some technical issues that initially limited accessibility of the PGR. When the PGR was launched for a patient, the message generated by the MyGeisinger portal was a default generic message that notified the recipient that a survey was available. This was confusing and led many patients to delete the message without accessing the report. A customized message has been developed to solve this issue. Another problem was when the intended recipient was a caregiver of the patient, the caregiver had to have proxy access to the MyGeisinger portal to receive the message and launch the PGR. Proxy access is now addressed during the clinical encounter where the results are reported and caregivers are given instructions to create proxy access.

While the tool has been developed as part of a larger project involving whole genome sequencing, the tool could be used to supplement traditional genetic test reports as well. The tool also has the potential to support large scale sequencing projects with return of results. Geisinger is partnering with the Regeneron Genetics Center to perform exome sequencing on as many as 250,000 Geisinger patients over the next 5 years [17]. Over 90,000 exomes are already completed and analysis to identify pathogenic variants in genes that have been determined to have clinical actionability (such as *BRCA 1* and *2, LDLR,* etc.) [18]. The PGR is being used to support the clinical reporting of actionable genomic findings. As of December 2017, over 500 clinical reports have been issued. PGRs have been issued for 61 participants and their providers with the remaining 500 prioritized for release over the next 3 months. Going forward, the clinical reporting process has been modified such that GPRs will be issued when the participant is contacted. The large number of reports will allow robust evaluation and improvement. In addition, a pharmacogenomic (PGx) PGR has been developed [19]. Because PGx results are quite different from causal Mendelian genetic variants, the PGx PGR was developed using the same end user engagement approach as the PGR described here and elsewhere. Additional use cases are being explored.

Demonstration of compatibility with other tools such as the SGPA enhances the utility and generalizability of the PGR tool. This implementation required a significant amount of effort from personnel at both sites as it required development of novel interfaces and customized local solutions. Standards-based approaches would be desirable to allow more rapid enhancements such as was used to incorporate OpenInfobutton functionality, which required very little developer time and no local customization. Recently, SMART on FHIR has been touted as a new approach that will enable “plug-in apps” to run natively inside any compliant EHR [20]. The HL7-developed FHIR standards are designed to support retrieval of data services needed to drive applications, while the SMART platform is designed to launch from the EHR and facilitate the launching of apps, all within extant clinical workflows. Combined with OAuth2 authorization, the SMART on FHIR platform provides a standards-based technology stack for app development. Geisinger is currently exploring usage of SMART on FHIR capabilities in platforms and applications including COMPASS™. Most relevant to the PGR, SMART on FHIR Genomics is emerging as a genomic-specific HL7 approved standard [21]. The feasibility of the prototype was demonstrated through incorporation in three representative applications based on the SMART platform. Recently, SMART on FHIR Genomics has been selected for use in the All of Us program (previously the Precision Medicine Initiative) through the Sync for Genes project [22]. Incorporating the functionality of SMART on FHIR Genomics into the PGR is being explored. As adoption of these standards increases, so will the potential scalability and portability of current and future web applications such as the PGR. 

## Conclusions

In conclusion, we believe that to realize the promise of genomic and ultimately precision medicine requires increased engagement with patients and their caregivers. The PGR represents the first effort to develop and implement a tool to engage patients and their providers within the EHR ecosystem. It addresses currently identified barriers and implements all the elements of the ONCHIT Self-care and Community domains [8].

## Abbreviations

API: Application Program Interface
EHR: Electronic Health Record
FHIR: Fast Healthcare Interoperable Resources
HTML: Hypertext Markup Language
MS SQL: Microsoft Structured Query Language
ONCHIT: Office of the National Coordinator of Health Information Technology
PGR: Patient-facing Genomic test Report
PGx: Pharmacogenomics
SGPA: Simulconsult® Genome Phenome Analyzer
SMART: Substitutable Medical Apps, Reusable Technology
SOAP: Simple Object Access Protocol
SQL: Structured Query Language
XML: eXtensible Markup Language
